# Early Immunomodulatory Effects of Different Natural Surfactant Preparations in Preterms With Respiratory Distress

**DOI:** 10.3389/fped.2022.845780

**Published:** 2022-03-18

**Authors:** Mehmet Yalaz, Sema Tanriverdi, Özgün Uygur, Özge Altun Köroğlu, Elif Azarsiz, Guzide Aksu, Nilgün Kültürsay

**Affiliations:** ^1^Department of Pediatrics, Division of Neonatology, Ege University Medical School, Izmir, Turkey; ^2^Department of Pediatrics, Division of Neonatology, Manisa Celal Bayar University Medical School, Manisa, Turkey; ^3^Department of Pediatrics, Division of Neonatology, Izmir Tepecik Training and Research Hospital, Izmir, Turkey; ^4^Department of Pediatrics, Division of Pediatric Immunology, Ege University Medical School, Izmir, Turkey

**Keywords:** surfactant, prematurity, respiratory distress syndrome, immune response, newborn

## Abstract

**Background:**

Respiratory distress syndrome (RDS) is the most common respiratory disease in premature infants. Exogenous natural surfactant preparations are used in the treatment of RDS. In recent years, it has become increasingly evident that surfactant plays an immunoregulatory role.

**Objectives:**

The aim of this study was to evaluate cytokine and chemokine response following three different regimens of natural surfactant treatment in preterm newborns with RDS.

**Methods:**

Premature newborns below 32 weeks of gestation who were intubated for RDS and given early surfactant rescue therapy were included in the study. Newborns were randomly divided into three groups and Beractant 100 mg/kg (B-100), Poractant alfa 100 mg/kg (Pα-100) and Poractant alfa 200 mg/kg (Pα-200) were administered intratracheally. Blood samples and transtracheal aspirates (TA) were collected just before and 4–6 h after the surfactant treatment. Total eosinophil count, inducible T Cell alpha chemoattractant (ITaC), macrophage inflammatory protein 3 beta (MIP3b), interleukins (IL) 5, 8, 9, 10, 13, immunoglobulin E (IgE), interferon gamma (IFN-γ), eotaxin and tumor necrosis factor beta-1 (TGF-β1) were measured from blood and tracheal aspirate samples.

**Results:**

A total of 45 infants, 15 in each group, were included in the study. Mean gestational age, birth weight, antenatal, demographic and clinical characteristics of the study groups were similar. IFNγ concentration and eosinophil counts in TA decreased after surfactant replacement in all groups, especially in the infants treated with Pα-100 and Pα-200. Eotaxin, TGF beta and IL-8 concentrations in TA increased significantly in the infants treated with Pα-100 and Pα-200. IL-9 levels in TA decreased in the B-100 group but increased in the Pα-100 and Pα-200 groups. Blood levels of cytokines and chemokines showed significantly decreased levels of ITaC and MIP3b only in the B-100 group, but no significant change was observed in the Pα-100 and Pα-200 groups.

**Conclusion:**

In our study, the different immunomodulatory effects of natural surfactant preparations on newborn lung is proven. We found that Poractant α, one of the natural surfactant preparations, shifted the lung immune system toward TH2.

## Introduction

Respiratory distress syndrome (RDS) is a disease that can result in serious morbidities and death in premature infants. It is mainly caused by alveolar surfactant deficiency accompanying structural immaturity in the lung. Surfactant, which is a phospholipid-protein mixture (90% lipids, 5–10% proteins), reduces the surface tension keeping the alveoli open (1, 2). It is well known that natural exogenous surfactant treatment reduces mortality and morbidity in premature infants with RDS (3). Natural exogenous surfactant preparations, which have been shown to be more effective in infants with RDS, should be preferred in the treatment. Natural exogenous surfactant preparations are obtained by bronchopulmonary lavage or from mincing the lung tissue and extraction with organic solvents; it can then be purified by chromatography or supplemented with active ingredients. All surfactant preparations contain all the phospholipids found in endogenous lung surfactant; surfactant protein B and C also form part of the chemical composition.

Poractant alpha (Poractant α), one of the natural surfactant preparations available in our country, is produced by the lipid separation and purification of minced pork lung tissue by liquid gel chromatography. Its phospholipid concentration is 76 mg/ml, and protein concentration is 1 mg/ml (0.45 mg/ml surfactant protein B and 0.55 mg/ml surfactant protein C). Another natural surfactant preparation, Beractant, is produced from minced bovine lung tissue by lipid cleavage, and supplemented with dipalmitoylphosphatidylcholine, palmitic acid and tripalmitin. Its phospholipid concentration is 25 mg/ml and protein concentration is <1 mg/ml (surfactant protein B and C). As is seen, both preparations exhibit differences per dose and content. However, surfactant A and D proteins are not present in preparations available in the market (4). In addition to their roles in surfactant homeostasis, surfactant A and D proteins are known to be crucial host defense components against respiratory pathogens and allergens, play immunomodulatory roles (5).

Surfactant is an important component of the innate and adaptive immune system of the lung. It is necessary to regulate the immune response of the lung (6). Knowledge on the immunomodulatory effects of natural exogenous surfactant preparations in newborns is limited. RDS is characterized by intense inflammation along with the influx of neutrophils, monocytes, and macrophages within the alveoli and their released cytokines and chemokines such as interleukin 1 (IL-1), interleukin 8 (IL-8), interleukin 12 (IL-12), interferon-gamma (IFN-γ) and tumor necrosis factor alpha (TNF-α). The increase in the concentrations of these cytokines and chemokines were associated with severity of RDS. This inflammation significantly damages the lung parenchyma (7, 8). These proinflammatory mechanisms may cause secondary surfactant inactivation (7, 9). *In vitro* studies showed that, in cell cultures that have been exposed to inflammatory stimuli, Poractant α suppressed TNF-α synthesis and TNF-α receptors (10), Beractant was found to inhibit IL-1 beta and suppress the synthesis of Macrophage inflammatory protein 1 (MIP-1) (11). *In vivo* studies have also confirmed that surfactant applied intratracheally suppresses cytokine release (12). In a study comparing two surfactant preparations of 200 mg/kg Poractant α and 100 mg/kg Beractant treatment doses, it was shown that it did not affect the airway inflammatory response and there was no significant difference between IL-6 and IL-8 values in the tracheal aspirate (13). In this study, cytokine and chemokine responses were investigated in preterm infants with RDS following three different natural surfactant treatment protocols.

## Materials and Methods

Premature infants below 32 weeks of gestation who were intubated in our hospital for RDS and were treated with early surfactant rescue were included in our study. Surfactant replacement therapy should be administered as soon as possible (within 1–2 h after birth) in infants with RDS symptoms and a need for surfactant, which is the current strategy recommended for lung protection (1). The study was carried out with the approval of the Ege University Clinical Research Ethics Committee (12-1.1/15) and the financial support of the Ege University Scientific Research Projects Unit (MY-13-TIP-030).

Intubated premature infants with clinical findings of RDS were randomized according to their hospitalization protocol numbers and divided into three groups. Intubation was performed and intratracheal 100 mg/kg Beractant (B-100), 100 mg/kg Poractant alfa (Pα-100) and 200 mg/kg Poractant alfa (Pα-200) was administered to the infants in the 1st group, the 2nd group and the 3rd group, respectively.

Infants who needed mechanical ventilation for non-pulmonary conditions (congenital malformations such as cardiac defects, gastrointestinal defects such as abdominal wall defects and diaphragmatic hernia) were excluded from the study. Demographic and clinical features of newborns (intraventricular hemorrhage, patent ductus arteriosus, bronchopulmonary dyplasia, retinopathy of prematurity, pneumothorax, early and late onset sepsis, mortality) were recorded. During hospitalization, blood culture samples were obtained from the umbilical catheter and treated with ampicillin and gentamicin. Appropriate total parenteral fluid support was started, and minimal enteral nutrition was initiated for all newborn babies within the first 24 h of life.

In our neonatal unit, according to international guidelines, mechanical ventilation therapy was performed using the Babylog 8000 plus (Draeger, Lübeck, Germany) synchronized intermittent positive pressure ventilation-volume guarantee (SIPPV-VG) ventilation mode. Initial ventilator settings; were set to tidal volume (VT): 4 mL/kg, positive end-expiratory pressure (PEEP): 5 cm H_2_O, peak inspiratory pressure (PIP) 2–3 cm H_2_O higher than minimum pressure to achieve target VT, respiratory rate: 40–60/min, inspiratory time: 0.30-0.3 and fraction of inspired oxygen (FiO_2_) was adjusted to provide a saturation of 90–95%. Respiration rate, VT, and FiO_2_ were adjusted during weaning from the mechanical ventilator.

### Sample Collection

Blood and tracheal aspirate fluid samples were collected just before and 6 h after surfactant administration. Tracheal aspirate was taken from the endotracheal tube after 1 cc of saline was given and positive pressure was applied 5 times, and negative pressure was applied with a sterile aspiration catheter. Eosinophil counts from blood and tracheal aspirate samples were immediately done with an instant Abbott Cell DYN 3700 counter. Other tracheal aspirate fluid and blood samples were centrifuged at 10,000 rpm and the obtained samples were stored at −80°C until batch analysis. After all samples were collected, inducible T Cell alpha chemoattractant (ITaC), macrophage inflammatory protein 3 beta (MIP3b), eotaxin, IL-5, IL-8, IL-9, IL-10, IL-13, immunoglobulin E (IgE), IFN-γ, tumor necrosis factor beta-1 (TGF-β1) levels were measured by ELISA method in Ege University Medical Faculty Immunology Laboratory. The results of serum and tracheal aspirate samples in the three surfactant treatment protocols were compared. All cases in the study groups were monitored prospectively in terms of clinical findings, short term morbidity and mortality.

### Statistical Analysis

Statistical analysis was performed using the SPSS for Windows statistical package (SPSS version 21.0 for Windows, IBM Corp., US). The difference between two groups was examined by independent samples *t*-test for normally distributed variables and the Mann–Whitney U-test for non-normally distributed variables. Differences among more than two groups were examined by ANOVA with repeated measures. A paired *t*-test was used to compare the arithmetic averages of two independent groups. The chi-square was used to compare categorical variables between groups. The Kolmogorov–Smirnov test was used to evaluate the normal distribution assumption for numerical variables. A *p*-value of <0.05 was considered statistically significant.

## Results

Forty-five premature infants with clinical signs of RDS who were intubated were randomized into three groups. Fifteen preterm infants in group 1 were on B-100, 15 preterm infants in group 2 were on Pα-100, and 15 preterm infants in group 3 were on Pα-200. The mean gestational age of the groups (B-100 Group: 28.13 ± 2.55 weeks; Pα-100 Group: 28.80 ± 2.56 weeks; Pα-200 Group: 27.46 ± 3.64 weeks) and birth weights (B-100 Group: 1212.33 ± 382.82 grams; Pα-100 Group: 1319, 80 ± 390.95 grams; Pα-200 Group: 1127.07 ± 840.00 grams), demographic and antenatal characteristics showed no difference among the groups (Table 1).

**Table 1:** Comparison of demographic characteristics of the groups.

**Groups**	**B-100** ***(n = 15)***	**Pα-100** ***(n = 15)***	**Pα-200** ***(n = 15)***	***P*** **-value**
Gestational age (week) (mean ± SD)	28.13 ± 2.55 (25–32)	28.80 ± 2.56 (23–32)	27.46 ± 3.64 (22–32)	0.475
Birth weight (g) (mean ± SD)	1212.33 ± 382.82 (760–1940)	1319.80 ± 390.95 (800–1880)	1127.07 ± 840.00 (630–1880)	0.471
Cesarean section (*n*, %)	15 (%100)	13 (%86.7)	11 (%73.4)	0.099
Male gender (*n*, %)	8 (%53.4)	8 (%53.4)	7 (%46.6)	0.915
Antenatal steroid treatment^*^ (*n*, %)	7 (%46.6)	11 (%73.4)	10 (%66.6)	0.293
Chorioamnionitis^**^ (*n*, %)	1 (%6.6)	3 (%20)	5 (%33.3)	0.189
PROM^***^ (*n*, %)	5 (%33.3)	7 (%46.6)	7 (%46.6)	0.695
Apgar score at 1^st^ min (median)	5 (3–8)	5 (4–8)	5 (4–8)	0.586
Apgar score at 5^th^ min (median)	7 (6–10)	7 (6–10)	7 (6–10)	0.378

* *Antenatal steroid therapy was considered to be given if mother received two doses of 12 mg of betamethasone intramuscularly 24 h apart at any time prior to delivery*.

** *Chorioamnionitis was diagnosed by if two or more of the following were present: maternal fever equal to or higher than 37.8°C, with no alternative source; white blood cell count above 15,000 cells/mm3; uterine fundal tenderness; fetal tachycardia and vaginal discharge with purulent or foul odor*.

*** *PROM, premature rupture of membranes*.

There was no difference in terms of the hours of surfactant administered as early rescue therapy among the groups. There was no significant difference among the three groups with regard to mechanical ventilation need, mechanical ventilation duration, oxygen index (OI), nasal continuous positive airway pressure (nCPAP), and pneumothorax frequency (Table 2).

**Table 2:** Comparison of respiratory follow-up of the groups.

**Groups**	**B-100** ***(n = 15)***	**Pα-100)** ***(n = 15)***	**Pα-200)** ***(n = 15)***	***P*** **-value**
Mechanical ventilation duration (day) (mean ± SD)	14.07 ± 19.00 (1–70)	10.40 ± 14.21 (1–57)	35.26 ± 64.59 (2–260)	0.209
Oxygen index (before)	6.89 (10.8)	8.5 (7.5)	8.6 (15.1)	0.454
Oxygen index (after)	5.76 (3.6)	5.6 (12.4)	6.14 (3.3)	0.315
nCPAP^*^ duration (day) (mean ± SD)	9.60 ± 9.20 (1–21)	11.71 ± 11.54 (2–33)	12.25 ± 13.25 (1–39)	0.922
Oxygen treatment duration (day) (mean ± SD)	22.76 ± 26.24 (1–80)	21.06 ± 36.37 (1–149)	47.86 ± 69.46 (4-280)	0.257
Pneumothorax (*n*, %)	2 (%13.3)	3 (%20)	6 (%40)	0.209
Surfactant administration time after birth (hour)	1.53 (1.12)	1.93 (1.33)	2.07 (1.43)	0.513

* *nCPAP, Nasal Continuous Positive Airway Pressure*.

In the clinical follow-up of the cases, no significant difference was found among the groups in terms of sepsis, patent ductus arteriosus (PDA), retinopathy of prematurity (ROP), necrotizing enterocolitis (NEC), bronchopulmonary dysplasia (BPD), length of hospital stay and mortality. However, intraventricular hemorrhage (IVH) and hydrocephalus rates were found to be significantly higher in group 3 (Table 3).

**Table 3:** Comparison of short morbidity and mortality of the groups.

**Groups**	**B-100** ***(n = 15)***	**Pα-100** ***(n = 15)***	**Pα-200** ***(n = 15)***	***P*** **-value**
Hemodynamically significant PDA^*^ (*n*, %)	6 (%40)	8 (%53.3)	7 (%46.6)	0.765
BPD^**^( ***n*** **, %)**				0.185
None	9 (%60)	11 (%73.3)	8 (%53.3)	
Mild	3 (%20)	2 (%13.3)	0 (%0)	
Moderate	0 (%0)	0 (%0)	2 (%13.3)	
Severe	3 (%20)	2 (%13.3)	5 (%33.3)	
BPD^**^-postnatal steroid usage (*n*, %)	3 (%20)	5 (%33.3)	5 (%33.3)	0.548
Pulmonary hypertension (*n*, %)^***^	1 (%6.6)	4 (%26.6)	3 (%20)	0.345
Pulmonary hemorrhage (*n*, %)	2 (%13.3)	4 (%26.6)	3 (%20)	0.659
**Sepsis (** ***n*** **, %)**				0.624
None	3 (%20)	1 (%6.6)	2 (%13.3)	
Clinical	9 (% 60)	10 (%66.6)	7 (%46.6)	
Proven	3 (%20)	4 (%26.6)	6 (%40)	
**Early sepsis (** ***n*** **, %)**				0.175
None	7 (%46.6)	4 (%26.6)	3 (%20)	
Clinical	8 (%53.3)	11 (%73.3)	10 (%66.6)	
Proven	0 (%0)	0 (%0)	2 (%13.3)	
**Late sepsis (** ***n*** **, %)**				0.827
None	7 (%46.6)	8 (%53.3)	6 (%40)	
Clinical	6 (%40)	5 (%33.3)	7 (%46.6)	
Proven	2 (%13.3)	2 (%13.3)	2 (%13.3)	
ROP^****^( ***n*** **, %)**				0.823
No	13 (%86.6)	12 (%80)	12 (%80)	
Yes	2 (%13.3)	3 (%20)	3 (%20)	
ROP^****^-laser treatment (*n*, %)	0 (%0)	1 (%6.6)	1 (%6.6)	0.518
IVH^*****^( ***n*** **, %)**				**0.027**
None	10 (%66.6)	5 (%33.3)	4 (%26.6)	
Stage 1	2 (%13.3)	4 (%26.6)	1 (%6.6)	
Stage 2	0 (%0)	1 (%6.6)	0 (%0)	
Stage 3	0 (%0)	4 (%26.6)	8 (%53.3)	
Stage 4	3 (%20)	1 (%6.6)	2 (%13.3)	
Hydrocephalus (*n*, %)	3 (%20)	2 (%13.3)	8 (%53.3)	**0.046**
NEC^******^( ***n*** **, %)**				0.933
None	9 (%60)	7 (%46.6)	8 (%53.3)	
Stage 1	1 (%6.6)	2 (%13.3)	2 (%13.3)	
Stage 2	2 (%13.3)	3 (%20)	1 (%6.6)	
Stage 3	3 (%20)	3 (%20)	4 (%26.6)	
NEC^******^ surgery (*n*, %)	3 (%20)	3 (%20)	3 (%20)	1
Mortality (*n*, %)	7 (%46.6)	7 (%46.6)	6 (%40)	0.914
Length of hospital stay (day) (mean ± SD)	31.08 ± 31.64 (1-107)	40.27 ± 46.83 (1-149)	58.40 ± 68.55 (4-280)	0.373

* *PDA, patent ductus arteriosus*;

** *BPD, bronchopulmonary dysplasia;*

**** *ROP, retinopathy of prematurity;*

***** *IVH, intraventricular hemorrhage;*

****** *NEC, necrotizing enterocolitis*.

*** *Pulmonary hypertension was diagnosed in the patients with clinical signs by pediatric cardiology echocardiography. The bold values are statistically significant*.

**Figure 1:**
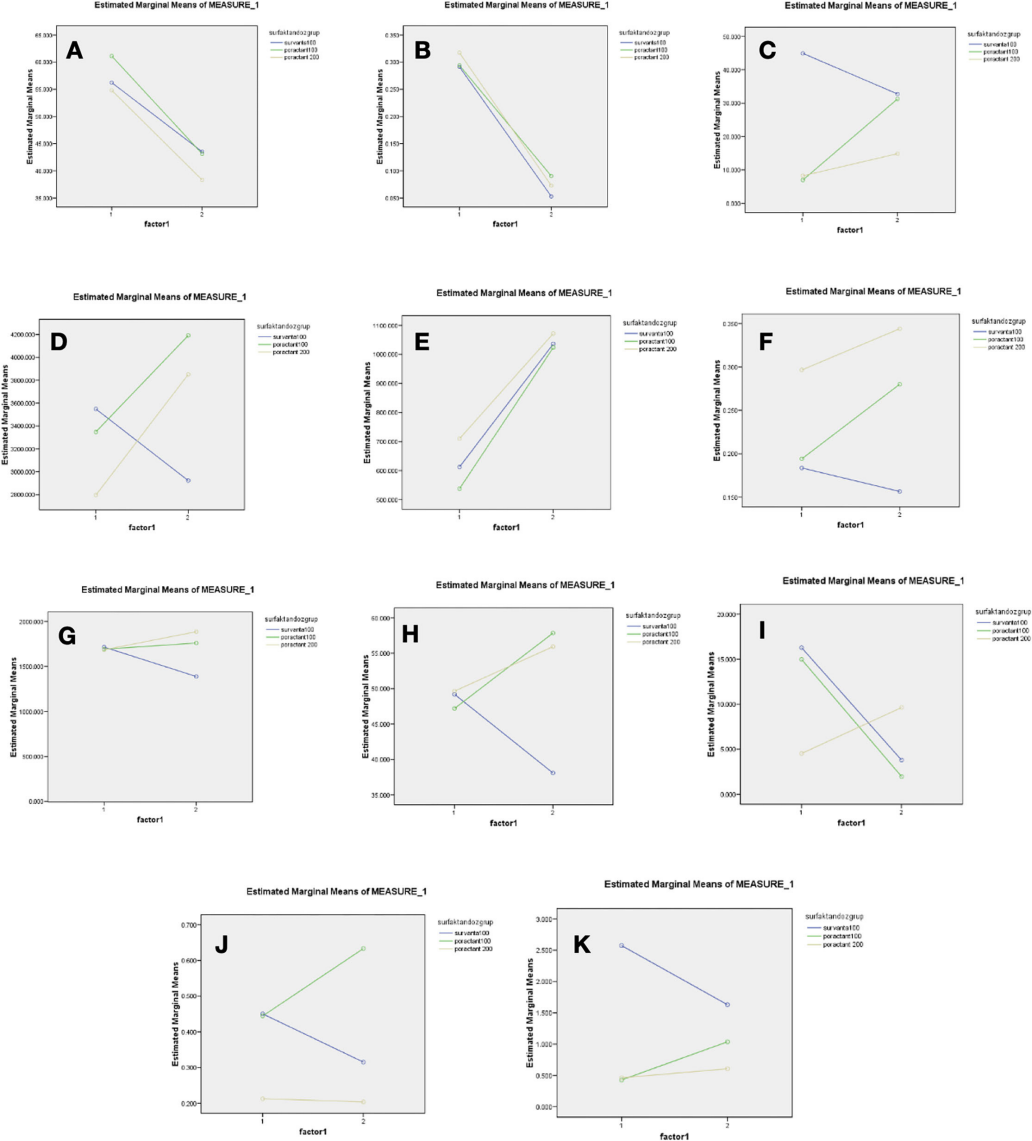
Levels of intratracheal chemokines and cytokines before and after surfactants administration in all three groups **(A)** TA [Eosinophil (%)] **(B)** TA (IFN-γ) **(C)** TA (8Eotaxin) **(D)** TA (TGF beta) **(E)** TA (IL-8) **(F)** TA (IL-9) **(G)** TA (ITaC) **(H)** TA (MIP) **(I)** TA (IgE) **(J)** TA (IL-5) **(K)** TA (IL-10).

Chemokine and cytokine responses of three surfactant treatment protocols (B-100, Pα-100 and Pα-200) in preterm infants with RDS were evaluated in tracheal aspirate (TA) samples before and after surfactant administration (Table 4). Eosinophil ratios and IFN-γ levels decreased significantly in three groups after surfactant therapy (*p* < 0.05). Eotaxin and TGF-β1 levels decreased significantly in the B-100 group after surfactant, and eotaxin and TGF-β1 levels increased significantly in the Pα-100 and Pα-200 groups (*p* < 0.05). IL-8 levels increased significant ly in all three groups after treatment (*p* > 0.05). There were no significant changes in ITaC, MIP3b, IgE, IL-5, IL-9, IL-10, IL-13 levels in the three groups (Figure 1).

**Table 4:** Comparison of cytokine and chemokine levels before and after surfactant in tracheal aspirate samples of three groups.

**Groups**	**B-100** ***(n** **=** **15)***			**Pα-100** ***(n** **=** **15)***			**Pα-200** ***(n** **=** **15)***		
	**Before**	**After**	***p-*** **value**	**Before**	**After**	***p-*** **value**	**Before**	**After**	***p-*** **value**
Eosinophil ratio (%)	57	44	*	62	43	*	55	38	*
IFN-γ (mean ± SD)	0.28 ± 0.14	0.05 ± 0.03	*	0.29 ± 0.14	0.07 ± 0.17	*	0.33 ± 0.01	0.09 ± 0.07	*
Eotaxin (mean ± SD)	44.92 ± 84.79	32.75 ± 50.03	*	6.98 ± 4.56	31.26 ± 29.03	*	8.25 ± 5.33	14.87 ± 8.11	*
TGF-β1 (mean ± SD)	3548.46 ± 1257.13	2922.58 ± 1670.26	*	3347.88 ± 1993.56	4190.92 ± 1950.93	*	2794.88 ± 1625.88	3850.17 ± 1793.02	*
IL8 (mean ± SD)	616.51 ± 840.69	1036.10 ± 784.12	*	541.66 ± 804.63	1024.01 ± 852.40	*	714.16 ± 862.73	1071.86 ± 869.02	*
IL9 (mean ± SD)	0.17 ± 0.13	0.15 ± 0.14	**	0.18 ± 0.14	0.22 ± 0.12	**	0.28 ± 0.14	0.32 ± 0.12	**
ITaC (mean ± SD)	1753.64 ± 615.40	1324.15 ± 292.75	**	1744.74 ± 462.51	1823.57 ± 302.96	**	1758.16 ± 691.23	1857.83 ± 607.60	**
MIP 3b (mean ± SD)	48.86 ± 64.98	38.08 ± 45.80	**	47.04 ± 54.58	57.84 ± 52.05	**	49.65 ± 47.95	55.92 ± 90.85	**
IgE (mean ± SD)	16.28 ± 5.92	3.80 ± 6.10	**	14.97 ± 4.77	3.98 ± 2.35	**	4.52 ± 7.73	9.60 ± 2.09	**
IL5 (mean ± SD)	0.467 ± 0.076	0.328 ± 0.077	**	0.458 ± 0.058	0.680 ± 0.077	**	0.211 ± 0.076	0.210 ± 0.077	**
IL10 (mean ± SD)	0.258 ± 0.261	1.741 ± 0.143	**	0.322 ± 0.229	1.038 ± 0.227	**	0.423 ± 0.183	0.630 ± 0.214	**
IL13 (mean ± SD)	16.97 ± 50.67	15.72 ± 46.06	**	14.43 ± 26.89	14.43 ± 35.50	**	15.58 ± 60.40	18.34 ± 17.81	**

^*^p < 0.05; ^**^p > 0.05.

Chemokine and cytokine responses of three surfactant treatment protocols (B-100, Pα-100, Pα-200) in preterm infants with RDS were evaluated in blood serum samples before and after surfactant administration (Table 5). Only ITaC and MIP3b levels showed a significant decrease in the B-100 group, while no significant change was seen in the Pα-100 and Pα-200 groups (Table 5).

**Table 5:** Comparison of cytokine and chemokine levels before and after surfactant in blood samples of three groups.

**Groups**	**B-100** ***(n** **=** **15)***			**Pα-100** ***(n** **=** **15)***			**Pα-200** ***(n** **=** **15)***		
	**Before**	**After**	***p-*** **value**	**Before**	**After**	***p-*** **value**	**Before**	**After**	***P-*** **value**
IFN- γ (mean ± SD)	0.52 ± 1.26	0.57 ± 0.88	**	0.41 ± 0.78	0.60 ± 0.89	**	0.66 ± 1.14	0.47 ± 027	**
Eotaxin (mean ± SD)	71.186 ± 47.70	58.17 ± 20.65	**	86.053 ± 51.52	87.06 ± 47.74	**	50.59 ± 29.25	58.53 ± 43.20	**
TGF-β1 (mean ± SD)	29583.00 ± 9111.39	24914.20 ± 3942.51	**	28950.20 ± 15095.30	27513.40 ± 15619.05	**	26928 ± 12708.01	21018.21 ± 11875.05	**
IL8 (mean ± SD)	684.11 ± 537.26	718.77 ± 570.61	**	847.88 ± 688.37	1012.96 ± 571.47	**	600.83 ± 429.86	696.35 ± 462.32	**
IL9 (mean ± SD)	1.26 ± 0.30	1.25 ± 0.24	**	1.29 ± 0.32	1.30 ± 0.22	**	1.35 ± 0.65	1.36 ± 0.58	**
ITaC (mean ± SD)	1715.14 ± 927.30	1388.29 ± 805.12	*	1691.85 ± 1039.44	1760.46 ± 1017.68	**	1685.58 ± 1024.27	1785.66 ± 1086.92	**
MIP3b (mean ± SD)	165.53 ± 43.18	138 ± 30.95	*	172.57 ± 55.46	170.52 ± 58.87	**	191.72 ± 53.24	232.58 ± 116.61	**
IgE (mean ± SD)	12.30 ± 3.10	12.43 ± 4.30	**	13.77 ± 4.28	13.48 ± 4.88	**	13.11 ± 2.47	13.25 ± 3.24	**

^*^p < 0.05; ^**^p > 0.05.

## Discussion

Our results show that natural exogenous surfactant administration in preterm infants with RDS may cause some quantitative changes in local and systemic immune mediators which may have a possible short and/or long-term effects, although we were unable to predict long-term outcomes. Pulmonary surfactant is a lipoprotein complex that performs two main functions. It reduces surface tension at the air-liquid interface of the lung and it plays a role in host defense against infection and inflammation (14). The literature of the last 20 years has revealed that endogenous pulmonary surfactant has immunoregulatory abilities that can alter the function of the innate and acquired immune system (15). Surfactant has the potential to interact with a variety of immune system cells that regulate allergen- or pathogen-induced airway inflammatory events. Surfactant modulates airway physiology and immunology in inflammatory conditions (6).

The main risk factors for pulmonary pathophysiological sequelae from RDS in premature infants include immature lung tissue, decreased host defense and antioxidant systems, volutrauma and barotrauma due to mechanical ventilation, oxidant damage and infection from supplemental oxygen exposure (16). A common finding among these risk factors is that they are known to cause an undesirable inflammatory response in the lung. Events that lead to inflammation in the lung and subsequent remodeling of the lung structure result in the inflammatory response immediately after birth even if lung protective respiratory strategies are used (17). Surfactant-treated preterm lambs and baboons with RDS develop BPD associated with elevation of proinflammatory cytokines in the lungs (18, 19). These findings have also been reported in newborn infants who subsequently developed BPD (20, 21). These relationships support the claim that inflammation in the lung is an important precursor in the development of BPD (16).

Surfactant consists of four different types of proteins: SP-A, SP-B, SP-C and SP-D. The hydrophilic proteins i.e., SP-A and SP-D are members of the collectin protein family that have the ability to opsonize pathogens and facilitate phagocytosis by macrophages and monocytes (22). The immunoregulatory property of surfactant is provided by surfactant proteins A and D (23). They regulate the production of inflammatory mediators (24). At the onset of allergic inflammation, SP-A and SP-D inhibit T lymphocyte proliferation (25). Interestingly, SP-A increased TGF-β secretion and increased the frequency of regulatory T lymphocytes (26). Both SP-A and SP-D inhibit IgE binding to allergens, suppress histamine release from mast cells, suppress TH2 cells and decrease TH2 cytokine levels, increase TH1 cytokine level, and stimulate anti-inflammatory TGF-β1 secretion (27).

In recent years, SP-A and SP-D have attracted more and more attention because of their role in host defense and lung immune responses (23). In experimental asthma models, it has been shown that acute allergic airway inflammation is more severe as a result of increased eosinophil infiltration and abnormal activation of lymphocytes, which leads to the production of TH2 cytokines in SP-A or SP-D deficient mice (28). On the contrary, inflammatory cytokines released during allergic inflammation lead to impaired pulmonary surfactant function (29, 30). It has been shown that administration of synthetic surfactant preparations (without SP A and SP-D) in asthmatic patients improves lung function; however it also worsens the underlying allergic immune response (6).

Recent studies have shown that phospholipids and hydrophobic proteins SP-B and SP-C, which are essential for biophysical functions, also have anti-inflammatory and antibacterial functions and contribute to immunomodulation (31, 32). It has been demonstrated that SP-B and SP-C reduce the activation of neutrophil granulocytes, can also affect other immunological cells such as macrophages, and reduce lipopolysaccharide-induced inflammation via their anti-inflammatory properties (33, 34). It has been found that SP-C binds to lipopolysaccharide through its N-terminal shadow and contributes to immunomodulation as a result of its effect (31, 32, 35). CHF5633, a new synthetic surfactant preparation containing SP-B and SP-C analogs, has been shown to reduce lipopolysaccharide-induced TNF-α and IL-1 beta cytokine production in human neonatal monocytes (36).

It was reported that SP-B deficiency results in reversible pulmonary inflammation and leads to neutrophil migration to the lung (37). Mice lacking SP-C have an excessive and persistent immune response of macrophages to viral and bacterial lung infections, suggesting that SP-C is a factor that minimizes inflammation (35). Unlike SP-A and SP-D, SP-C does not opsonize pathogens, while the absence of SP-C results in impaired phagocytosis by alveolar macrophages (38).

Natural and synthetic surfactant preparations have been used successfully in the treatment of RDS (39). Natural surfactant preparations contain all the phospholipids found in endogenous lung surfactant, SP-B and SP-C proteins; however, SP-A and SP-D proteins are lacking (40).

Surfactant proteins of natural surfactant preparations, phospholipids of natural and synthetic surfactant preparations, as well as artificial components are antigenic (41). Whether there is a risk of immunological sensitization in infants administered exogenous surfactant is still a matter of debate. Furthermore, surfactant and its components modulate the initiation of the inflammatory response by changing the immune function of the lung (42). *In vitro* studies have documented an interaction between alveolar macrophages and surfactant in immunological defense during inflammatory processes in the lungs. It has been suggested that the change in surfactant phospholipids in various pathological conditions may cause an increase in macrophage accumulation in the alveolar space (43). The mechanisms by which exogenous surfactants or phospholipids modulate the inflammatory cascade in alveolar macrophage are unclear (44). Probably cytokines play a central role in the immune response and pathogenesis (45). It has been shown that synthetic surfactant reduces the level of inflammatory cytokines in human alveolar macrophages and suppresses cytokine production in TH1 (46).

In the study conducted in preterm lambs, the synthetic surfactant lucinactant (the amount of KL4 peptide similar to SP B in its structure is 3 times higher than that of Beractant and Poractant α) showed that lung and systemic inflammation was less severe, and lung structural integrity was preserved compared to natural animal-derived Beractant and Poractant α (47). It is a key component of SP-B's ability to reduce alveolar surface tension, maintain surfactant structure, and protect against lung injury (48). These protective effects increase with the higher concentration of SP-B-like KL4 peptide in lucinactant (47). CHF5633, a new generation synthetic surfactant containing SP-B and SP-C synthetic analogs, is a surfactant preparation that also has no proinflammatory effect, balances pro- and anti-inflammatory cytokine response (49). Bezerra et al. (50) showed that proinflammatory markers IL 17 and TNF-α were reduced after the administration of Poractant α to adult mice with ARDS. Van Rensburg et al. (51) compared the immunogenic effects of three surfactant preparations (synthetic surfactant Synsurf, natural porcine lung surfactant Poractant α and bovine lung surfactant Liposurf in which the phospholipid concentrations were standardized as 500–1,500 μg/ml. They found that the proinflammatory markers IL-8, TNF-α and IFN-γ levels were significantly decreased in the bronchoalveolar lavage samples taken after administration of all three surfactant preparations in sick children. It was stated that there was a significant increase in the levels of anti-inflammatory markers IL-10 and IL-12. These changes were more pronounced in the Poractant α group (51).

In our study, when the lung inflammatory response to intratracheal exogenous natural surfactant preparation was evaluated in intubated preterm infants with RDS, as proinflammatory markers IFN-γ decreased significantly, IL-8 increased significantly while no significant changes were observed in the levels of anti-inflammatory markers IL-10 and IL-13 in tracheal aspirate samples examined before and after the application of porcine and bovine lung surfactant. It was seen that Pα-100, Pα-200, and B-100 natural surfactant preparations had no significant proinflammatory and anti-inflammatory effects in the lung.

However, it was seen that eosinophil ratio, IFN-γ, eotaxin and TGF-β1 levels decreased significantly, and IL-8 levels increased significantly in tracheal aspirate samples after B-100 administration. Eosinophil ratio and IFN-γ level decreased significantly, and eotaxin, TGF-β1 and IL8 levels increased significantly after administration of both 100 mg/kg and 200 mg/kg doses of Poractant α. As an important marker of TH1 response, IFN-γ level decreased, while no significant change was observed in the level of ITaC, which was measured to evaluate chemotaxis only after Pα-100 and Pα-200 treatment. On the other hand, IL-8 level, which is an indicator of inflammation and oxidative stress and a TH2 marker, increased significantly. Besides the level of eotaxin, which evaluates eosinophil migration and allergic response increased significantly. All these findings suggested a TH2 shift as the immunological response to Poractant α. The IL-9 level, which evaluates bronchial hyperreactivity, was also insignificantly elevated in the Poractant α group. There was no consistent change in IL-5 and IL-10 and IL-13 cytokine levels, which were measured to evaluate the TH2 response.

One of the pathophysiological mechanisms of asthma is associated with the immunomodulatory properties of surfactant (52). It has been shown that Poractant α given after allergy provocation in asthmatic patients accelerates eosinophilic infiltration, eotaxin and IL-5 cytokine levels, which indicate the TH2 response in the bronchoalveolar lavage fluid, increase, and IFN-α levels decrease (53). Dry powder surfactant (Pumactant) inhalation administered after allergen provocation in patients with allergic asthma abolished the early phase response of asthma, but did not affect the late phase response (54). In a study comparing natural porcine, bovine and synthetic surfactants in *in vitro* studies, Poractant α showed higher eosinophil chemotaxis compared to other surfactant preparations, but lower eosinophil viability, more eosinophil necrosis and apoptosis, and higher cytokine levels, eosinophilic cationic protein (ECP) eotaxin released from eosinophils (53, 55). These findings indicate that the therapeutic surfactant, especially Poractant α, may affect allergic inflammation in the lung by acting on eosinophils (56). When the interaction between TH2 and surfactant proteins were investigated in animal studies, it has been shown that SP-D levels are significantly higher in the lungs of mice with high IL-4, IL-5, IL-13 cytokine levels (57, 58). SP-D, which is not found in natural surfactant preparations, has been shown to be an immune molecule that plays a protective role against allergies, asthma and inflammation. In experimental studies, it has been shown that SP-D inhibits the allergen-IgE interaction, suppresses histamine release from mast cells and specific IgE production. It can also shift the TH2 response to the TH1 response in allergic stimulation. Interestingly, it is known that Surfactant D deficient mice have excessive IL-13 secretion and IL-13 plays a role in the development of asthma (59).

It is known that exogenous surfactant treatment reduces mortality and morbidity in premature infants with RDS. In studies comparing surfactant treatment with placebo, it has been shown that the severity of RDS, mortality, mid-long-term pulmonary morbidities such as BPD, and pneumothorax are reduced. When poractant alfa is administered at an initial dose of 200 mg/kg, it has been shown to be associated with lower mortality than the same preparation at a dose of 100 mg/kg or beractant 100 mg/kg. However, it is not clear whether this relationship is dependent on the dose or the application of different surfactant preparations (3, 60, 61). In our study, the clinical follow-up of the cases, no significant difference was found among the groups in terms of BPD, pneumothorax and mortality.

## Conclusion

In conclusion, natural surfactant preparations continue to be used successfully in the treatment of RDS in the neonatal period, but it is still a matter of debate whether there is a risk of immunological sensitization in patients given surfactant preparations. In our study, we found that Poractant α, one of the natural surfactant preparations, shifted the lung immune system toward TH2, which made us think that due to this immunomodulatory feature of surfactant, it may predispose to allergic inflammatory conditions such as asthma and bronchial hyperreactivity in older ages. SP-A and SP-D, which are not found in natural surfactant preparations, are known to reduce allergic inflammation and bronchial hypersensitivity. Therefore, the biological importance of Surfactant protein A and D against various lung diseases is increasingly recognized. The use of surfactant preparations supplemented with SP-A and SP-D in the treatment of RDS, which is the most common lung disease in preterm infants, seems to be promising due to early or late-term immune response regulation that may occur in the lung.

### Limitations

The size of the population sample is limited. We did not compare immunological response to synthetic surfactants in this study.

## Data Availability Statement

The original contributions presented in the study are included in the article/supplementary material, further inquiries can be directed to the corresponding author/s.

## Ethics Statement

The studies involving human participants were reviewed and approved by Ege University Clinical Research Ethics Committee (12-1.1/15). Written informed consent to participate in this study was provided by the participants' legal guardian/next of kin. Written informed consent was obtained from the minor(s)' legal guardian/next of kin for the publication of any potentially identifiable images or data included in this article.

## Author Contributions

All authors listed have made a substantial, direct, and intellectual contribution to the work and approved it for publication.

## Funding

This study was carried out with the financial support of the Ege University Scientific Research Projects Unit (MY-13-TIP-030).

## Conflict of Interest

The authors declare that the research was conducted in the absence of any commercial or financial relationships that could be construed as a potential conflict of interest.

## Publisher's Note

All claims expressed in this article are solely those of the authors and do not necessarily represent those of their affiliated organizations, or those of the publisher, the editors and the reviewers. Any product that may be evaluated in this article, or claim that may be made by its manufacturer, is not guaranteed or endorsed by the publisher.
